# Immunoproteomic and mass spectrometric analysis of *Eimeria acervulina* antigens recognized by antisera from chickens infected with *E. acervulina*, *E. tenella* or *E. necatrix*

**DOI:** 10.1186/s13071-020-3965-y

**Published:** 2020-02-21

**Authors:** Jin Liu, Wenbin Tuo, Xiangdong Wu, Jiaming Xiong, Enchao Yu, Chao Yin, Zhiwu Ma, Liheng Liu

**Affiliations:** 1 Jiangxi Provincial Key Laboratory for Animal Health, College of Animal Science and Technology, Jiangxi Agricultural University, Nanchang, 330045 Jiangxi People’s Republic of China; 2 Animal Parasitic Diseases Laboratory, Agricultural Research Service, United States Department of Agriculture, Beltsville, MD 20705 USA

**Keywords:** *Eimeria*, Sporozoite, Common immunoreactive antigens, Two-dimensional gel electrophoresis (2-DE), Immunoproteomics, Mass spectrometry

## Abstract

**Background:**

Coccidiosis is caused by *Eimeria* spp. and can result in severe economic losses to the global poultry industry. Due to anticoccidial drug resistance rapidly developing in the parasites and drug residues in poultry products, efficacious and safe alternative coccidia control measures are needed. The objective of the present study was to identify common protective antigens which may be used as vaccine candidates in the development of subunit, multivalent, cross-protective vaccines against most of the economically important *Eimeria* species.

**Methods:**

Whole sporozoite proteins of *Eimeria acervulina* were prepared and analyzed by 2-dimensional gel electrophoresis (2-DE) followed by western blotting using immune sera specific to *E. tenella*, *E. acervulina*, or *E. necatrix*. The protein spots detected by all three immune sera were then excised from the preparative gel and protein ID was performed by MALDI-TOF-MS/MS.

**Results:**

Approximately 620 *E. acervulina* sporozoite protein spots were demonstrated by 2-DE with silver staining, among which 23 protein spots were recognized by immune sera specific to all three *Eimeria* species. The results showed that 21 putative *E. acervulina* proteins were identified, which include proteins with known enzymatic properties, and those which are involved in protein translation, transport and trafficking, and ribosomal biogenesis and functions. There is one protein which may be involved in transcription and one heat-shock protein. Two proteins contain predicted domains, but with no apparent functions known. There were 2 protein spots which had no detectable proteins. None of the proteins has a predicted signal peptide or a transmembrane domain; however, 6 of the 21 putative proteins were predicted to be potentially secretory through the non-classical pathway.

**Conclusions:**

Our study identified a diverse group of antigens immunologically common to all three *Eimeria* species, none of which was previously characterized and tested as a vaccine candidate. Further research on immunogenicity and cross-protective potential of these individual proteins as vaccine candidates will aid the development of vaccines against the most common and pathogenic *Eimeria* spp.
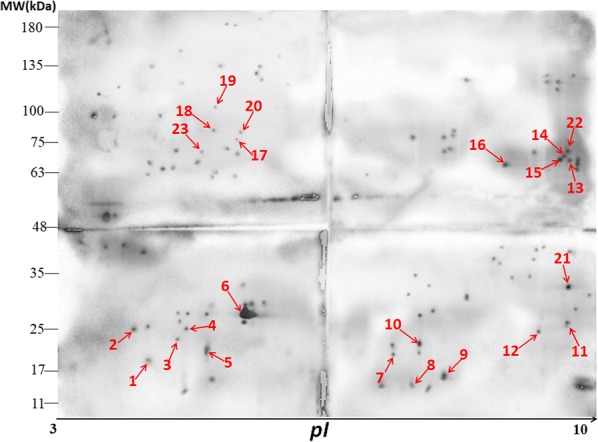

## Background

Poultry coccidiosis is an intestinal disease caused by the protozoan parasites of the genus *Eimeria* [1]. It has been estimated that coccidiosis can cause more than €2 billion in annual losses to the global poultry industry [2]. The infection in chickens usually results in intestinal bleeding, reduced feed conversion, weight loss, and even death in severe cases [3]. *Eimeria* species have a complicated life-cycle with 3 major stages: schizogony, gametogony and sporulation [4]. It has been well documented that there are multiple pathogenic *Eimeria* species, including *E. necatrix*, *E. tenella* and *E. acervulina* [5, 6]; thus, they are the focus of the present study.

The current control methods of poultry coccidiosis primarily rely on the use of anti-coccidian drugs and/or live attenuated vaccines [7, 8]. However, there are two main problems with these methods, the emergence of drug resistant strains and drug residues in poultry products [9]. The current use of the live attenuated *Eimeria* strains as vaccines still has to face the risk of residual pathogenesis to the birds, reversion to pathogenic forms and high cost of producing and maintaining the live vaccines for the manufacturers and producers. Therefore, subunit coccidiosis vaccines, produced using recombinant DNA technology and having the advantage of being safe, cost-effective and efficacious, are urgently needed. In recent years, DNA-based vaccines against coccidiosis have been reported with some protection [10–14]. However, it is well known that field coccidiosis is often caused by co-infections with multiple pathogenic strains of *Eimeria* [15, 16]. Hence, it is necessary to develop a vaccine that cross-protects the chickens from most of the pathogenic strains of *Eimeria*.

Proteomics has been successfully performed to study and identify drug targets in parasites [17, 18]. Furthermore, immunoproteomics has been used to identify the antigens of *Eimeria* origin. de Venevelles et al. [19] performed immunoproteomics on identification of immunoreactive proteins in *E. tenella* sporozoites, which resulted in discovery of approximately 50, out of a total of 130, proteins recognized by anti-*E. tenella* sera. Two-dimensional gel electrophoresis (2-DE) and western blotting were used to analyze the second-generation merozoite proteins of *E. tenella*, where approximately 640 proteins were demonstrated on the proteome map and 85 of them were recognized by the anti-*E. tenella* sera [20]. In addition, Zhang et al. [21] also conducted immunoproteomics studies with *Eimeria* antigens and identified 46 proteins as antigens of the unsporulated *E. tenella* oocysts.

In the present study, whole sporozoite proteins of *E. acervulina* were prepared and analyzed by 2-DE and western blotting. *Eimeria acervulina* protein spots recognized by anti-sera against *E. tenella*, *E. acervulina*, and *E. necatrix* were collected and analyzed for protein ID by mass spectrometry. A total of 620 *E. acervulina* sporozoite protein spots were identified by 2-DE and silver staining, 21 of which were identified as antigens immunoreactive to hyperimmune sera of all three species of *Eimeria*. These 21 immunologically common proteins are putative, uncharacterized previously and highly diverse in cellular locations and predicted functions. Further research on these shared antigens may lead to the development of cross-protective, multivalent vaccines against coccidiosis.

## Methods

### Animals

One-day-old San Huang chicks (Wenzhen Hatchery, Nanchang, China) were raised under coccidia-free conditions before the infection experiments.

### Parasites

Oocysts of *E. acervulina*, *E. necatrix* and *E. tenella* (Nanchang strains) were stored in our laboratory. Oocysts of the three *Eimeria* species were propagated, harvested and sporulated as reported previously [22, 23]. The sporulated oocysts were stored in 2.5% (w/v) potassium dichromate solution at 4 °C no longer than 2 weeks. Sporozoite isolation was conducted, as described previously, using a DE-52 anion exchange column for purification [24]. The purified sporozoites were stored in liquid nitrogen before use.

### Protein sample preparation

The purified sporozoites of the *E. acervulina* were suspended with lysis buffer [7 M urea, 2 M thiourea, 4% (w/v) CHAPS, 1% (w/v) DTT, 1% protease inhibitor cocktail (v/v) and 2% (v/v) immobilized pH gradient buffer] and sonicated in ice bath (200 W, work time 6 s, interval time 15 s, for 60 cycles). Soluble proteins were obtained after centrifugation at 15,000× *rpm* for 10 min at 4 °C. The supernatants were treated with a 2-D clean-up kit and protein concentrations were quantified using PlusOne™ 2-D Quant Kit (GE Healthcare Life Sciences, Pittsburgh, PA, USA) [20, 25].

### Two-dimensional gel electrophoresis (2-DE)

#### Isoelectric focusing (IEF)

IEF was performed as previously reported [20, 26]. To achieve increased separation of proteins, 24 cm IPG strips (pH 3–10, non-linear; GE Healthcare Life Sciences) were used for IEF. Briefly, the protein preparation was mixed with immobilized pH gradient (IPG) rehydration/sample buffer [8 M urea, 2% CHAPS, 20 mM DTT, 0.5% (v/v) IPG buffer, pH 3–10, and 0.001% bromophenol blue] and incubated for at least 1 h at room temperature. Sporozoite proteins (200 µg) were loaded onto an IPG strip followed by rehydration for 12 h at 20 °C by using rehydration buffer. IEF was performed using preset programs on EttanIgphor II (GE Healthcare Life Sciences) in 4 steps: S1 at 0–50 V for 12 h; S2 at 50–8000 V for 4 h; S3 at 8000–10000 V for 4 h; and S4 at 10000 V for 4 h.

#### Sodium dodecyl sulphate polyacrylamide gel electrophoresis (SDS-PAGE)

Prior to SDS-PAGE, the gel strips were incubated in equilibration buffer I (50 mM Tris-HCl, pH 8.8, 6 M urea, 2% SDS, 30% glycerol and 1% DTT) for 15 min and then in equilibration buffer II (50 mM Tris-HCl, pH 8.8, 6 M urea, 2% SDS, 30% glycerol and 2.5% iodoacetamide) for 15 min. The strips and SDS-PAGE molecular weight standards (11–180 kDa; Thermo Fisher Scientific, Waltham, MA, USA) were then loaded onto the 12.5% polyacrylamide gels [21]. Each sample was simultaneously run on 2 separate gels, one was used for western blotting, the other was used for silver staining, according to the methods reported previously [27]. ArtixScan 1010 Plus (Microtek International, Inc., Taiwan, China) was used for imaging gels.

#### Analysis of images

The stained gels were analyzed using the ImageMaster™ 2D Platinum Software (Version 5.0, GE Healthcare, San Francisco, CA, USA) for protein spot detection, quantification and matching as well as comparative and statistical analyses [26].

### Antiserum preparation

Chickens were raised coccidia-free until 2 weeks-old, and then divided into 4 groups (*n* = 30) and housed in 4 separate rooms. Three groups were orally infected with sporulated oocysts of *E. tenella*, *E. acervulina* or *E. necatrix.* The dose of first infection was 1 × 10^5^ sporulated oocysts per chicken [28]. Three days after the first infection, the chickens were infected 4 more times, at 3-day intervals, at a dose of 5000 sporulated oocysts per chicken. The remaining group of chickens was used as negative control, and inoculated by gavage with distilled water only. Five weeks after the last infection, blood samples were obtained from chicken’s wing vein, coagulated for 1 h at 37 °C, and then incubated overnight at 4 °C. After centrifugation at 4000× *rpm* for 10 min, the serum was collected, aliquoted, and stored at − 20 °C until used [29]. Specific antibody titers were determined in 2-fold serial dilutions by ELISA using *E. acervulina* sporozoite antigens. All sera used in this study had ELISA titers of 1280 or higher.

### Western blot

To increase transfer efficiency, each gel was cut into 4 equal pieces prior to transfer. Proteins were transferred to a polyvinylidene fluoride (PVDF) membrane of 0.45 μm pore size (GE Healthcare). Membranes were blocked with 5% non-fat-dried milk in phosphate-buffered saline (PBS) (pH 7.4) containing 0.05% Tween 20 (PBST) for 2 h at room temperature with shaking. Membranes were then incubated with one of the hyper-immune sera (1:100) for 2 h at room temperature. The pooled uninfected chicken sera were used as a negative control. After washing with PBST, the membrane was incubated with a secondary reagent (1:2000, donkey anti-chicken IgY conjugated with horseradish peroxidase; Proteintech Group, Inc., Rosemond, IL, USA) for 2 h at 37 °C. The membranes were washed with PBST for 1 h and developed with an enhanced chemiluminescence detection kit (GE Healthcare Life Sciences). The ChemiDoc™ XRS + with Image Lab™ Software (Bio-Rad, Hercules, CA, USA) was used for imaging and image analysis [26].

### Mass spectrometric (MS) analysis of protein spots, database search and protein sequence analysis

As previously reported, protein spots were removed from the SDS-PAGE gel and subjected to trypsin digestion and desalination [30]. MS analysis of protein spots was performed by the Experimental Center of Nanjing Medical University (Nanjing, China). MS and MS/MS data for protein ID were obtained by using a MALDI-TOF-TOF instrument (Bruker Daltonics, Bremen, Germany). The database search was conducted against the non-redundant protein sequences (nr) of NCBI (NCBInr) using the Mascot search engine (http://www.matrixscience.com). The search parameters included taxonomy of other Alveolata, one missed trypsin cleavage site allowed, fixed modifications of carbamidomethyl (C), variable modifications of oxidation (M), 100 parts per million mass accuracy, and MS/MS fragment tolerance set to 0.4 Da. The results of the search were considered positive if Mascot score of a protein is greater than 71 (*P* < 0.05) and sequence coverage is more than 15% with at least 4 matching peptides [31]. Protein orthologs within the three species of *Eimeria* were further compared for sequence identity using the multiple sequence alignment program CLUSTAL Omega (https://www.ebi.ac.uk/Tools/msa/clustalo/). Non-classical secretory property (SecP) and presence of a signal peptide (SP) and transmembrane domains were analyzed by the SecretomeP 2.0a Server (http://www.cbs.dtu.dk/services/SecretomeP/), SignalP-5.0 Server (http://www.cbs.dtu.dk/services/SignalP/) and TMHMM Server v. 2.0 (http://www.cbs.dtu.dk/services/TMHMM/), respectively.

## Results

### *Eimeria acervulina* sporozoite protein 2-DE profile

**Fig. 1:**
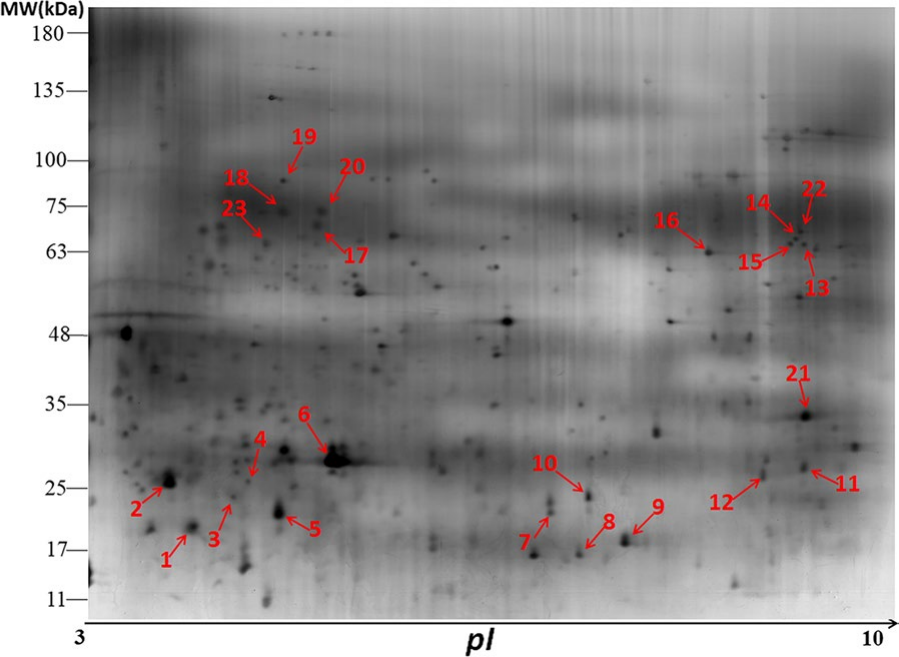
*Eimeria acervulina* sporozoite protein 2-dimensional gel electrophoresis (2-DE) profile demonstrated by silver staining. The soluble sporozoite proteins (200 μg) of *E. acervulina* were first resolved by isoelectric focusing (IEF) using a 24 cm immobilized pH gradient (IPG) strip (pH 3–10). Then, sodium dodecyl sulfate polyacrylamide gel electrophoresis (SDS-PAGE) (12.5%) was used to separate the proteins by size followed by silver staining. The Y-axis shows the protein Mr. range between 11 and 180 kDa and X-axis indicates the protein pI range between 3 and 10. Data represent results from 2 independent experiments

Analysis by 2-DE followed by silver staining revealed 620 protein spots in the soluble proteins of *E. acervulina* sporozoites. Most protein spots were located between 11 and 180 kDa (Fig. 1).

### Detection of common immunoreactive spots by western blot using immune sera from the three *Eimeria* spp

**Fig. 2:**
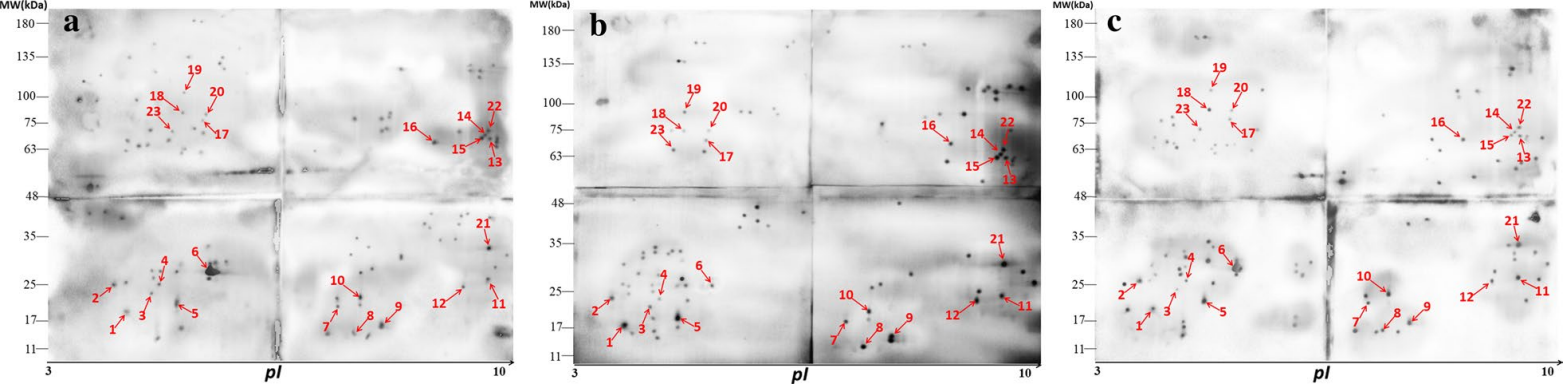
Detection of *Eimeria acervulina* sporozoite antigens separated with 2-dimensional gel electrophoresis (2-DE) by western blot using chicken anti-*E. acervuline* (**a**), anti-*E. necatrix* (**b**), or anti-*E. tenella* (**c**) sera. The proteins recognized by chicken immune sera against all three *Eimeria* species are indicated. Data represent results from 2 independent experiments

**Fig. 3:**
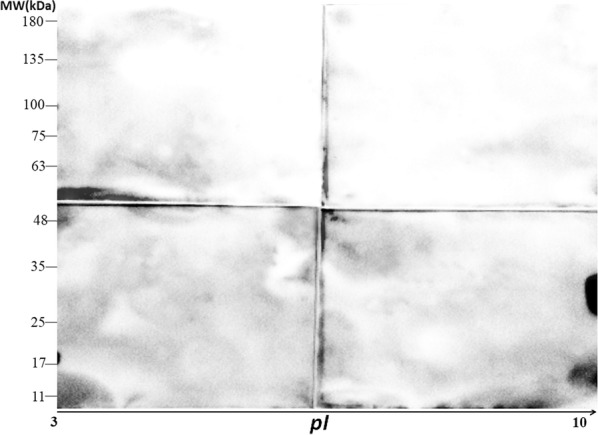
Western blot analysis of the 2-dimensional gel electrophoresis (2-DE)-separated *Eimeria acervulina* sporozoite antigens using pooled sera from negative control chickens. The control chickens were age-matched to those infected by *Eimeria* and raised separately under identical conditions. Data represent results from 3 independent experiments

Western blot analysis of 2-DE-separated *E. acervulina* sporozoite proteins using 3 individual immune sera showed that 118 protein spots were recognized by anti-*E. acervulina* serum, 92 protein spots were recognized by anti-*E. necatrix* serum, and 102 protein spots were recognized by anti-*E. tenella* serum. There was a total of 23 immunoreactive protein spots recognized by all three anti-*Eimeria* sera (Fig. 2, Table 1). No immunoreactive protein spots were detected by sera from negative control chickens (Fig. 3).

**Table 1:** Immunoproteomic and mass-spectrometric identification of *Eimeria acervulina* sporozoite proteins recognized by immune sera of the three *Eimeria* species studied

Spot ID^a^	Identified protein grouped by functions	Accession number^b^	No. of matched peptides^c^	Theoretical Mr/pI	Protein score^d^	Protein sequence coverage (%)^e^
Protein translation						
1	Elongation factor 1-beta, putative, partial	XP_013248731.1	16/40	19289/4.51	209	62
6	Eukaryotic translation initiation factor 3 subunit 8, putative	XP_013250573.1	14/37	31924/9.76	172	55
Transcription						
8	Elongin c, putative	XP_013249709.1	11/47	17120/5.34	144	49
Cargo transport and protein trafficking						
2	Intraflagellar transport particle protein, putative, partial	XP_013246990.1	16/46	28884/8.35	220	43
9	AP-4 complex subunit sigma-1, putative	XP_013248843.1	11/33	17189/5.41	135	40
Ribosome-related proteins						
7	60S ribosomal protein L8, putative	XP_013249139.1	12/40	28042/10.71	120	37
18	Ribosome biogenesis protein brix, putative	XP_013248150.1	17/54	54150/9.82	170	38
Predicted domain-containing proteins						
3	Uncharacterized ACR, YagE family COG1723 domain-containing protein, putative	XP_013250980.1	18/45	20048/9.58	240	49
4	U1 like C2H2 zinc finger, related	XP_013248237.1	14/35	28894/9.60	199	68
Chaperone proteins						
17	DnaJ domain-containing protein, putative	XP_013253345.1	15/49	48311/6.56	140	36
Protein of enzymatic properties						
5	Nucleolar GTP-binding protein, putative	XP_013252320.1	11/41	21119/9.70	147	57
10	Glycerol-3-phosphate dehydrogenase, putative, partial	XP_013248247.1	17/31	35465/6.67	237	60
11	Protein phosphatase 2C, putative	XP_013248554.1	13/56	42702/5.82	116	28
12	Glyceraldehyde-3-phosphate dehydrogenase, putative	XP_013251305.1	16/46	36753/7.57	211	41
13	Cysteine desulfurase, putative	XP_013251976.1	17/34	63362/8.43	191	45
14	Glyoxalase, putative	XP_013247421.1	20/39	65222/6.63	246	37
15	Phosphatase, putative	XP_013251081.1	14/38	62536/9.36	158	34
16	Phosphoribosylpyrophosphate synthetase, putative	XP_013248652.1	17/40	47500/9.30	171	57
19	CAM kinase, CDPK family TgPK1, putative	XP_013250395.1	15/36	118100/7.12	134	16
20	DNA photolyase, putative	XP_013251340.1	14/31	52839/8.79	147	37
21	gamma-glutamyl phosphate reductase, putative	XP_013252670.1	12/34	44087/5.46	153	36

^a^Protein spot numbering in the 2-DE gel; spots 22 and 23 that had no detectable protein signals are not listed

^b^Accession number in NCBI

^c^Number of peptides that match the predicted protein sequence

^d^Protein score is − 10*Log(P), where P is the probability that the observed match is a random event. Protein scores greater than 71 are significant (*P* < 0.05)

^e^Percentage of predicted protein sequence covered by matched peptides

### Protein identification and sequence analysis of the antigens recognized by immune sera specific to all three species of *Eimeria*

Peptide mass-fingerprinting (PMF) data of 23 protein spots were obtained by analysis with MALDI-TOF-MS/MS. The PMF data were then searched against the NCBInr using the Mascot search engine. Twenty-one *Eimeria* proteins with shared immunogenicity identified from protein spots 1 through 21 are summarized in Table 1. There were additional 2 spots (spots 22, 23) which were recognized by all three *Eimeria* sera (Figs. 1, 2), but no protein signals were detected (data not shown). The results showed that all the proteins identified in this study met the three criteria in that Mascot score is greater than 71, sequence coverage is more than 15%, and there are at least 4 matching peptides for each protein identified. In addition, sequence identity for the protein orthologs in all three *Eimeria* species, protein secretory property, and presence of signal peptides and transmembrane domains were analyzed and the results are summarized in Table 2.

**Table 2:** *Eimeria* protein amino acid sequence comparison

Spot ID^a^	Accession number	Amino acid sequence identity (%)^b^			SecP/SP/TM prediction^c^
		*E. acervulina*	*E. tenella*	*E. necatrix*^d^	
1	XP_013248731.1	100	58.72	44.88	0.053/N/N
2	XP_013246990.1	100	51.65	52.07	0.656*/N/N
3	XP_013250980.1	100	38.95	41.18	0.068/N/N
4	XP_013248237.1	100	48.54	48.71	0.371/N/N
5	XP_013252320.1	100	75.00	–	0.533/N/N
6	XP_013250573.1	100	75.19	75.19	0.843*/N/N
7	XP_013249139.1	100	95.14	94.33	0.462/N/N
8	XP_013249709.1	100	61.07	62.09	0.798*/N/N
9	XP_013248843.1	100	89.58	85.42	0.435/N/N
10	XP_013248247.1	100	73.67	73.35	0.42/N/N
11	XP_013248554.1	100	50.00	51.80	0.662*/N/N
12	XP_013251305.1	100	94.10	92.63	0.635*/N/N
13	XP_013251976.1	100	66.54	66.73	0.404/N/N
14	XP_013247421.1	100	58.44	60.60	0.245/N/N
15	XP_013251081.1	100	80.95	81.28	0.172/N/N
16	XP_013248652.1	100	84.56	89.52	0.799*/N/N
17	XP_013253345.1	100	96.00	96.00	0.283/N/N
18	XP_013248150.1	100	61.90	62.67	0.423/N/N
19	XP_013250395.1	100	61.68	47.85	0.298/N/N
20	XP_013251340.1	100	58.02	59.36	0.376/N/N
21	XP_013252670.1	100	67.26	67.51	0.454/N/N

^a^2-dimensional gel electrophoresis (2-DE) gel spot number

^b^All protein sequences within the same row are compared to that of *E. acervulina* using CLUSTAL Omega (https://www.ebi.ac.uk/Tools/msa/clustalo/)

^c^Non-classical secretory property (SecP) and presence of a signal peptide (SP) and transmembrane domains (TM) were analyzed by the SecretomeP 2.0a Server (http://www.cbs.dtu.dk/services/SecretomeP/), SignalP-5.0 Server (http://www.cbs.dtu.dk/services/SignalP/) and TMHMM Server v. 2.0 (http://www.cbs.dtu.dk/services/TMHMM/), respectively

^d^*Eimeria necatrix* ortholog for XP_013252320.1 was not found

*Abbreviations*: *, a SecP score exceeds the threshold (0.6), indicative of a secretory potential through the non-classical pathway for proteins with no signal peptides; N, not present

## Discussion

Identifying and testing common antigens as vaccine candidates across various *Eimeria* species is an immune response-based approach in the development of subunit vaccines against coccidiosis [32]. The advantage of using a cross-protective subunit vaccine is relatively safe, cost-effective, and labor-saving. Researchers have previously explored the common antigens of *Eimeria* species. Talebi [33] discovered at least one conserved protein band (45 kDa) from five *Eimeria* species (*E. acervulina*, *E. maxima*, *E. necatrix*, *E. praecox* and *E. tenella*), which was recognized by anti-*E. maxima* serum. Constantinoiu et al. [34] found that the apical complex of six species of *Eimeria* sporozoites (*E. brunetti*, *E. mitis*, *E. maxima*, *E. necatrix*, *E. praecox* and *E. tenella*) could be recognized by the monoclonal antibody 8E-1 using immunofluorescence analysis. Liu et al. [29] identified immunodominant proteins of three *Eimeria* species (*E. tenella*, *E. maxima* and *E. acervulina*), in which five groups of the protein orthologs were detected as common antigens. The *E. acervulina* 14-3-3 antigen was shown to induce significant immune responses and provided protection against *E. tenella*, *E. acervulina* and *E. maxima* infection [35]. The results of the present study added more putative, common, immunoreactive proteins to the list of cross-protective antigens, which should aid in facilitating the development of multivalent vaccines against co-infections by pathogenic *Eimeria* species.

The present study identified a group of proteins which are highly diverse in predicted cellular localizations and functions. Comparison of the amino acid sequences of orthologs showed that some antigens have moderate amino acid sequence conservation (spots 1, 3, 4 and 19). We speculate that, while most of the 21 common antigens may have both linear and conformational epitopes, some may reply heavily on conformational epitopes due to limited selections/availability of linear epitopes. Further, the lack of sequence identity may also be caused by incomplete protein sequences. Additional work is warranted to further confirm the correct annotation of the orthologs which appear to have large variations, having less than 50% in sequence identity with at least one of the three species in the genus (spots 1, 3, 4 and 19). No signal peptides or transmembrane domains were predicted from all 21 proteins, suggesting that none of the proteins are membrane-bound proteins or secretory proteins through the classical secretory pathway. Indeed, six proteins were predicted to be potentially secreted *via* the non-classical secretory pathway.

Only 1–2 proteins were identified in each of the following categories, including those with functions in protein translation, RNA transcription, cargo/protein transport and trafficking, ribosome function and biogenesis, and chaperone (heat-shock protein). Interestingly, immunization with the translation elongation factor EF-2 identified by 2-DE and MALDI in *Brugia malayi* adult worm protected the host from challenge infection with third-stage larvae [36]. Furthermore, a study using malaria-protective IgG identified a member of the heat-shock protein family, the eukaryotic translation initiation factor 3, and a ribosome-associated protein, which were considered vaccine candidates [37]. The only transcription factor identified in the present study was elongin C, which is essential for viral pathogenesis and replication, but there is no evidence that this protein may play a key role in protozoan pathogenesis [38]. Very little information is available about protozoan ribosome-related and HSP proteins demonstrated in this study.

The most striking finding of the present study is that 11 out of the 21 immunologically related antigens in all three *Eimeria* species are proteins of known enzymatic activities. In addition, five out of the 11 proteins with predicted enzymatic functions had been shown in other species that they can be vaccine candidates. These five proteins include glycerol-3-phosphate dehydrogenase (GPD, XP_013248247.1), protein phosphatase 2C (PP2C, XP_013248554.1), glyceraldehyde-3-phosphate dehydrogenase (GAPDH, XP_013251305.1), cysteine desulfurase (CDS, XP_013251976.1), and glyoxalase (GLO, XP_013247421.1). *Candida albicans* surface protein GPD binds to complement regulators for immune evasion by inactivating the complement cascade [39]. *Toxoplasma gondii*, an apicomplexan protozoan parasite, PP2C was identified as one of the highly upregulated genes *in vivo* by screening the *T. gondii* cDNA phage expression library using specific IgM and IgG [39]. The presence of upregulated immunogenic PP2C suggests that PP2C is a potential vaccine candidate for toxoplasmosis [40]. As a common antigen of *Eimeria*, GAPDH could induce strong humoral and cellular immune responses, and had effective protection against *E. tenella*, *E. acervulina* and *E. maxima* as a potential vaccine candidate [41]. In our present study, GAPDH as an immunodominant antigen was confirmed to be immunologically shared by all three *Eimeria* species. An epitope (a peptide called P39) of the GAPDH was identified on the *Plasmodium* sporozoite surface, and *Plasmodium* GAPDH was shown to bind to CD68 on the Kupffer cells [42]. Furthermore, the antibody to P39 suppressed sporozoite liver invasion without cross-recognizing host GAPDH [42]. In a study of bacterial surface proteomics, cysteine desulfurase, traditionally an intracellular protein, was identified on the bacterial cell surface. This protein and others characterized in that study were collectively named as “moonlighting proteins”, indicating that intracellular proteins found on cell surface are not as rare as we may anticipate [43]. A putative *Mycobacterium tuberculosis* glyoxalase was identified as an intracellular and a secretory protein and high percentage of human patients had anti-glyoxalase antibodies [43]. Most importantly, the increased levels of glyoxalase and the immunoregulatory cytokine interleukin-10 in serum positively correlates, suggesting that glyoxalase can be a potential vaccine target [44]. The other five proteins in the same group had little information available, and thus, it is difficult to estimate their potential as vaccine candidates. However, based on the reported findings that many nuclear or intracellular proteins are “moonlighting” on the cell surface or being secreted to the outside of the cells, the proteins common to all three *Eimeria* species that stimulated antibody responses should be considered potential vaccine targets [45, 46]. Ongoing research is testing for protective efficacies of the proteins identified in the present study, with formulations of either single protein vaccines or multivalent vaccines. The present study represents the first to show *E. acervulina* antigens recognized by sera specific to *E. acervulina* as well as those specific to *E. tenella* or *E. necatrix*. Interestingly, most of the *E. acervulina* proteins with shared immunogenicity identified in the present study were not found in a similar study where anti-*E. acervulina* sera were used to detect *E. acervulina* antigens [30]. One apparent difference between the studies is that we used antisera with much higher titers (1:1280), which could in part account for why the proteins identified in our study are not shown in the study by Liu et al. [30]. Future research is warranted to compare *Eimeria* antigens identified under similar experimental conditions.

## Conclusions

In this study, whole sporozoite proteins of *E. acervulina* were prepared and analyzed by 2-DE and western blotting. *Eimeria acervulina* protein spots recognized by three individual anti-sera against *E. tenella*, *E. acervulina* or *E. necatrix* were characterized. Twenty three out of 620 sporozoite protein spots were identified and 21 immunoreactive antigens common to all three species of *Eimeria* were identified, none of which was previously characterized and tested as vaccine candidates. Further research on immunogenicity and cross-protective potential of these individual proteins will aid in development of vaccines against the most common and pathogenic *Eimeria* spp.

## Data Availability

The datasets supporting the findings of this article are included within the article. The mass spectrometry proteomics data have been deposited to the ProteomeXchange Consortium *via* the PRIDE partner repository (http://www.ebi.ac.uk/pride) with the dataset identifier PXD016418.

## Abbreviations

2-DE: two-dimensional gel electrophoresis
MALDI-TOF-MS/MS: matrix-assisted laser desorption/ionization-time of flight-mass spectrometry/mass spectrometry
IPG: immobilized pH gradient
SDS-PAGE: sodium dodecyl sulphate polyacrylamide gel electrophoresis
MS: mass spectrometry
PMF: peptide mass fingerprinting
IEF: isoelectric focusing
GPD: glycerol-3-phosphate dehydrogenase
GAPDH: glyceraldehyde-3-phosphate dehydrogenase
CDS: cysteine desulfurase
GLO: glyoxalase
PP2C: protein phosphatase 2C
